# VISIBILITY OF STRUCTURES OF RELEVANCE FOR PATIENTS WITH CYSTIC FIBROSIS IN CHEST TOMOSYNTHESIS: INFLUENCE OF ANATOMICAL LOCATION AND OBSERVER EXPERIENCE

**DOI:** 10.1093/rpd/ncv556

**Published:** 2016-06-07

**Authors:** Carin Meltzer, Magnus Båth, Susanne Kheddache, Helga Ásgeirsdóttir, Marita Gilljam, Åse Allansdotter Johnsson

**Affiliations:** 1 Department of Radiology, Institute of Clinical Sciences, Sahlgrenska Academy at University of Gothenburg, SE-413 45 Gothenburg, Sweden; 2 Department of Radiology, Sahlgrenska University Hospital, SE-413 45 Gothenburg, Sweden; 3 Department of Radiation Physics, Institute of Clinical Sciences, Sahlgrenska Academy at University of Gothenburg, SE-413 45 Gothenburg, Sweden; 4 Department of Medical Physics and Biomedical Engineering, Sahlgrenska University Hospital, SE-413 45 Gothenburg, Sweden; 5 Gothenburg CF-Center, Sahlgrenska University Hospital, SE-413 45 Gothenburg, Sweden; 6 Department of Respiratory Medicine, Institute of Medicine, Sahlgrenska Academy at University of Gothenburg, SE-413 45 Gothenburg, Sweden

## Abstract

The aims of this study were to assess the visibility of pulmonary structures in patients with cystic fibrosis (CF) in digital tomosynthesis (DTS) using computed tomography (CT) as reference and to investigate the dependency on anatomical location and observer experience. Anatomical structures in predefined regions of CT images from 21 patients were identified. Three observers with different levels of experience rated the visibility of the structures in DTS by performing a head-to-head comparison with visibility in CT. Visibility of the structures in DTS was reported as equal to CT in 34 %, inferior in 52 % and superior in 14 % of the ratings. Central and peripheral lateral structures received higher visibility ratings compared with peripheral structures anteriorly, posteriorly and surrounding the diaphragm (*p* ≤ 0.001). Reported visibility was significantly higher for the most experienced observer (*p* ≤ 0.01). The results indicate that minor pathology can be difficult to visualise with DTS depending on location and observer experience. Central and peripheral lateral structures are generally well depicted.

## INTRODUCTION

Digital tomosynthesis (DTS) refers to the technique of collecting a number of low-dose projection radiographs at varying angles within a limited interval. A chest examination is typically based on 50–60 projection images acquired in the interval ±15° of the conventional posterior–anterior direction^(1–3)^. The projection images are then reconstructed to coronal section images, which provide an improved delineation of structures compared with that of conventional chest radiography (CXR), with reduced disturbances from overlying anatomy. The result is superior visibility of lesions that otherwise would be difficult or even impossible to identify^(4–11)^. DTS thereby provides some of the benefits of computed tomography (CT) at significantly lower radiation dose and cost, and shortened examination and reading times^(1–3)^. Currently, DTS is undergoing evaluation as a new diagnostic tool that might replace CT for certain tasks in thoracic radiology*,* for example characterisation of uncertain findings on CXR, and follow-up of pulmonary nodules^(12–15)^. Due to superior sensitivity to CXR and low radiation dose compared with CT, monitoring of cystic fibrosis (CF) lung disease has been suggested as another potential task for DTS^(2, 16)^.

The present study concerns imaging of lung pathology in patients with CF, which is a congenital and progressive disease affecting many organs. The structural pulmonary changes can lead to respiratory failure, which is the main cause of shortened life expectancy in this patient category. Early detection and treatment of airway infection and inflammation are crucial for preventing irreversible damage to the lung tissue and to delay disease progression^(17–19)^. Consequently, these patients require frequent and lifelong imaging surveillance from early childhood. Pulmonary changes related to CF have traditionally been radiologically monitored using CXR and CT. CXR offers low radiation dose, but is only moderately sensitive in depicting typical CF pathological lesions^(3, 9)^. CT is currently the gold standard for assessing lung parenchyma in CF since the modality is sensitive to both subtle structural changes and small areas with severe focal disease. Discrete pathology like thin-walled bronchiectasis observed in a CT image can be the first sign of CF, even when pulmonary function tests and blood samples are normal or unchanged^(18–21)^. However, a major concern regarding repeated CT examinations during childhood and adolescence is the potential risk of radiation-induced cancer^(21, 22)^, and reduction of radiation exposure is therefore of special importance for these individuals. DTS has been reported to expose the patient to an average of 0.13 mSv, which is considerably less than standard- and low-dose chest CTs (estimated to 3.0 and 0.5–1.5 mSv, respectively) and only two times more than a two-plane radiograph (estimated to 0.07 mSv)^(23)^. DTS has consequently emerged as an interesting ultra-low-dose alternative to CT.

Even though detection of pathology has been reported as significantly improved with DTS compared with CXR, limitations concerning depiction of structures adjacent to pleura, diaphragm, central vessels and mediastinum have been described^(9, 11)^. This is due to reduced depth resolution and susceptibility to motion for DTS in comparison with CT, which provides high resolution in all directions and faster image acquisition. However, to the knowledge of the authors, there is currently no published study that has assessed the visibility of pulmonary structures in DTS by carrying out a head-to-head comparison with CT, taking anatomical location and observer experience into account. Thus, the aims of the present study were to assess the visibility of specific pulmonary structures of relevance for patients with CF in DTS in comparison with CT, and to investigate the dependency on anatomical location and observer experience.

## MATERIALS AND METHODS

A prospective, non-blinded study was conducted. The Regional Ethical Review Board in Gothenburg, Sweden approved the study, and all participating patients gave oral and written informed consent.

### Patient characteristics

Adult patients with CF ranging from mild to severe, followed at Sahlgrenska University Hospital, were consecutively invited to the study, which involved an additional DTS in conjunction with the follow-up chest CT performed every 3 y. A total of 52 patients underwent the follow-up between March 2011 and February 2014, which also included lung function tests and blood samples. Of these, 21 patients underwent both helical CT and DTS on the same d, which was the inclusion criterion for the present study. Patient characteristics are listed in Table 1.

**Table 1.** Patient characteristics.

Characteristic	Number (%)	
Gender, women	11 (52)	
Pancreatic insufficiency	19 (90)	
Genotype F/F	14 (67)	
Port-a-cath (intravenous port)	10 (48)	
Breast implants	1 (5)	
	Median	Range
Age (y)	30	18–55
Weight (kg)	67.5	45.5–87.6
Length (cm)	175	150.5–189
BMI (kg/m^2^)	22.3	18.3–27
TLC (l)	6.4	3.8–10
TLC (% of predicted)	102.9	85–125
FEV1 (l)	2.4	1.3–5.2
FEV1 (% of predicted)	68	28–117.4

F = F508del-CFTR, the most common mutation in CF patients; BMI, body mass index (weight/height^2^); TLC, total lung capacity; FEV1, forced expiratory volume during the first second.

### Image acquisition

CT and DTS were performed within a time frame of 1.5 h. The DTS examinations were performed with the commercially available X-ray system GE Definium 8000, with the VolumeRAD option (GE Healthcare, Chalfont St Giles, UK). Low-dose projection radiographs were acquired in an upright position during a 10-s breath hold at full inspiration. The exposure setting used for the low-dose projections was determined by an initial scout image, acquired using automatic exposure control. The X-ray tube performed a movement in the caudocranial direction within ±15° of the standard posterior–anterior projection. The resulting 60 low-dose projections were reconstructed to ∼60 coronal section images with a reconstruction interval of 5 mm. The tube voltage was 120 kV. These acquisition parameters have been found optimal for chest tomosynthesis^(24)^.

A helical CT scan was performed in supine position and maximal inspiration, and a step-and-shoot CT scan was also performed at maximal expiration with one of the following CT scanners: LightSpeed Pro 16, LightSpeed VCT, Discovery CT750HD, Optima CT660 (GE Healthcare, Milwaukee, WI, USA) and Somatom Definition (Siemens Medical Solution, Forchheim, Germany). The inspiratory helical scan used as reference in the present study had a constant tube voltage of 120 kV, dose-modulated tube current in the range of 35–110 mA and a rotation time of 0.5 s. Transverse images with a slice thickness of 1–1.25 mm and 10 mm interspace, as well as coronal and sagittal images with a slice thickness of 4 mm and 1 mm overlap, were reconstructed from the helical series. All examinations were performed without intravenous contrast. The expiratory step-and-shoot images were not used in this study.

### Estimation of radiation dose

Effective dose for DTS was estimated by multiplying the dose-area product (DAP) of the DTS examination by the conversion factor of 0.26 mSv Gy^−1^ cm^−2 (25)^. The DAP of the DTS examination is not stored with the images, but was obtained by applying a validated method developed by Båth *et al.*^(26)^, where the DAP of the DTS examination is estimated from data stored in the digital imaging and communications in medicine (DICOM) header of the scout image. Effective dose for CT was estimated by multiplying the stored dose length product by the conversion factor of 0.017 mSv mGy^−1^ cm^−1^, as recommended by European guidelines^(27)^.

### Quality assessment of inspiration

Image quality assessment regarding adequate inspiration was performed visually. Additionally, the level of inspiration was quantitatively assessed on the frontal scout images of DTS and CT to assure that the examinations were comparable in this aspect. This method has been proposed^(28, 29)^ as a way of estimating total lung capacity (TLC) on chest radiographs. The estimated lung volume is based on the distance between the apex and pleural sinus of the right lung and the distance between the left and right pleural sinus.

### Definition of locations and structures in the reference CT scan for comparison of visibility

**Figure 1.**
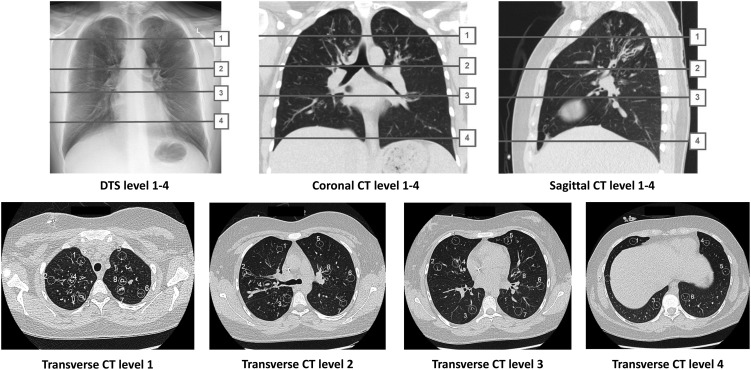
Illustration of the chosen anatomical Levels 1–4 on tomosynthesis and CT where Level 1 includes the right and left upper lobes, Level 2 the lower parts of the upper lobes and the apical parts of the lower lobes, Level 3 the middle lobe, lingula and central parts of the lower lobes and Level 4 the lower parts of the lower lung lobes.

In order to compare visibility according to anatomical location, structures in different regions of the lungs to be included in the study were defined in the reference transverse CT images. These were selected in order to include areas that are known to be problematic in DTS. With the intention of including structures from different parts of the lung parenchyma (lung lobes), four anatomical levels of interest were chosen. The first at the anterior insertion of the first rib, including the clavicle and in some cases intravenous devices (right and left upper lobes). The second at the level of the carina including the hilar region and also in some cases intravenous devices (lower parts of upper lobes, apical parts of lower lobes). A third level was determined, at the ostium of the lower pulmonary veins, including the heart and great vessels (middle lobe, lingula and lower lobes). Finally, the fourth basal level was placed where the diaphragm covers a great part of the field, in most cases approximately where the 11th ribs connect to the vertebral body (lower parts of lower lung lobes). The anatomical levels were termed 1–4 and are illustrated in Figure 1.

Specific regions of interest were selected at each anatomical level. At Levels 1–3, these included one central and three peripheral regions located in the anterior, posterior and lateral parts of the lung parenchyma. In each region, structures for assessment were identified by two of the authors in consensus (C.M. and Å.A.J.). Peripheral structures were defined as within 2 cm distance from the pleura. However, a relevant structure up to 2.5 cm from the pleura was accepted in the absence of other peripheral pathology. Level 4 had no central regions due to the close relation to the diaphragm, but three peripheral ones located in the anterior, posterior and lateral aspects of the visualised lung parenchyma.

**Figure 2.**
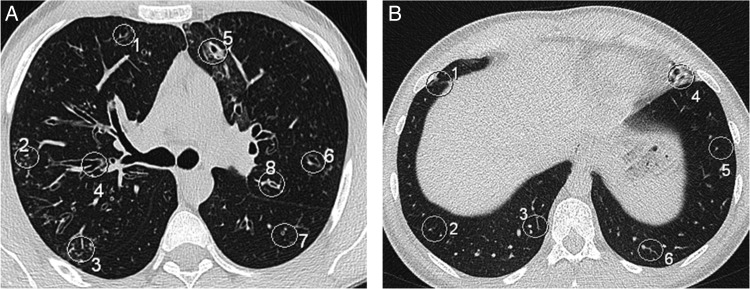
Representatives of analysed structures in different regions at two different levels. (**A**) Level 2 showing one central structure in each lung (Structures 4 and 8), and three peripheral structures located in the anterior, posterior and lateral parts of the lung parenchyma (Structures 1–3 and 5–7). (**B**) Level 4. The diaphragm covers a great part of the field, and three peripheral structures located in the anterior, posterior and lateral parts of the lung parenchyma were analysed (Structures 1–6).

Each structure of interest, for example a ‘peripheral mucus plug’, was marked with a circle in the transverse CT images, and briefly described in the study protocol to ensure that all observers assessed the same structure. The identified structures of interest were mainly bronchiectasis with and without bronchial wall thickening, mucus plugging and parenchymal consolidation. The majority of lesions were small, measuring ∼2–3 mm in diameter*.* In cases without pathological findings in the specific regions, the visibility of blood vessels was analysed instead. Blood vessels are often the only structure apart from lung tissue that can be visualised in CT images in the peripheral part of the lungs, and contrast and size are similar to small bronchioles with thickened walls often seen in CF. Examples of structures marked with circles are given in Figure 2.

### Observers

The visibility of structures predefined in the CT scan was individually assessed in DTS by three medical doctors with varying experience of CT, DTS and radiological findings of CF. Observer 1 (S.K.) was a thoracic radiologist with >25 y of experience in thoracic radiology including imaging of CF and 8 y of experience with DTS. Observer 2 (H.A.) was a specialist in pulmonary medicine with ∼1 month of full-time experience in DTS and CT, and some experience in clinical work with CF. Observer 3 (C.M.) was a resident radiologist with 2 y of experience in radiology including CT, and limited experience of DTS and CF. There was no training session prior to the study.

### Evaluation of visibility

The visibility of structures in the DTS images was assessed by a direct comparison with the appearance of the corresponding structure in the concurrently performed CT scan. First, the observers had to locate the anatomical structure defined in the transverse CT image in the DTS examination. During this process, the sagittal and coronal CT images were used for anatomical guidance. After identification of the corresponding anatomy and structure in the DTS examination, the observers compared the visibility of the structure with the reference visibility in the transverse CT image and gave their rating. Each structure was rated on a scale ranging from non-visible in DTS (0) to substantially improved visibility compared with CT (5). A description of the rating scale is given in Table 2.

**Table 2.** Rating scale of visibility of structures in DTS.

Grade	The structure seen in DTS is in comparison with CT assessed as being/having
0	Not visible
1	Barely visible
2	Visible, but with reduced details
3	Equal visibility
4	Improved visibility
5	Substantially improved visibility

### Statistical analysis

Statistical analysis was performed using visual grading characteristics (VGC)^(30, 31)^. VGC analysis is used in studies with ordinal data, which require non-parametric rank-invariant statistical methods, and produces a curve describing the relationship between two sets of data. The VGC curve is a plot of the proportion of ratings above a certain threshold for one set of data against the same proportion for another set of data at various threshold settings. The area under this VGC curve ranges from 0 to 1 and is used as figure of merit to describe the separation between the two datasets, an area of 0.5 indicating equality between the two datasets. In the present study, the area under the VGC curve described differences in perceived visibility between observers or between anatomical locations for a given observer. Results were calculated using VGC Analyzer^(32)^, based on the trapezoid VGC curve. A random-reader analysis was employed for comparisons of visibility according to anatomical location, in order to enable a generalisation of the results to the population of observers. Due to multiple comparisons in the analyses, a *p*-value of ≤0.01 was considered indicating statistical significance. The study also comprised descriptive analysis, as well as a Bland–Altman analysis^(33)^ for comparison of lung volume between the DTS and CT examination.

## RESULTS

In total, the study comprised 1890 visibility ratings based on 30 predefined anatomical structures in 21 patients who were evaluated by three observers. The estimated average effective dose was 3.1 mSv for helical CT (range 1.0–7.2) and 0.13 mSv for DTS (range 0.10–0.16). By visual inspection, the level of inspiration was judged as adequate on both DTS and CT for all patients. Mean estimated total lung volume was 0.6 1 larger in DTS than CT based on the scout images of both modalities (95 % limits of agreement ranged from −0.17 to 1.36).

### Overall visibility

**Figure 3.**
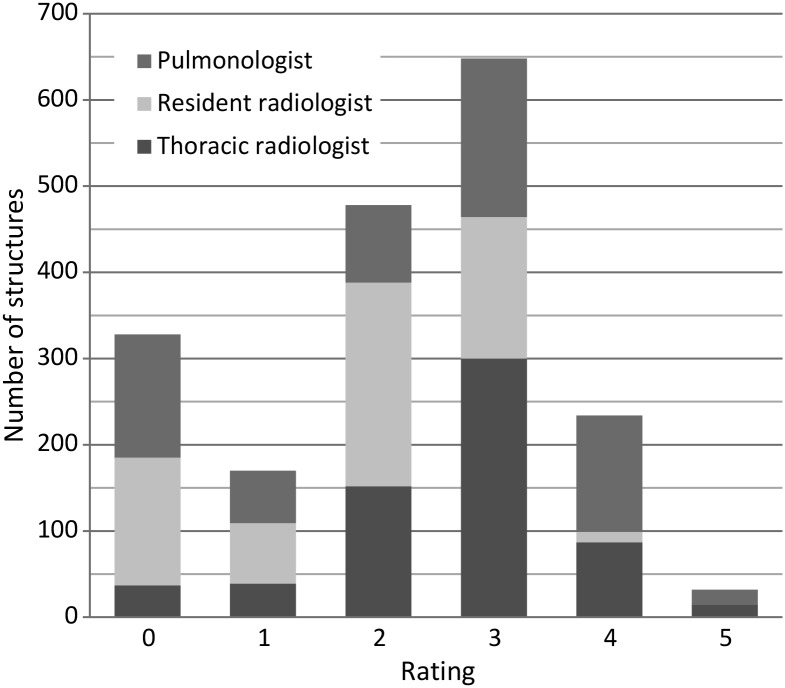
Histogram of ratings for all structures and observers.

The visibility in DTS compared with CT was reported as equal in 34 % (Grade 3), inferior in 52 % (Grades 0–2) and superior in 14 % (Grades 4 and 5) of the ratings. Perceived visibilities of all structures and observers are illustrated in Figure 3.

### Dependency on anatomical location

**Figure 4.**
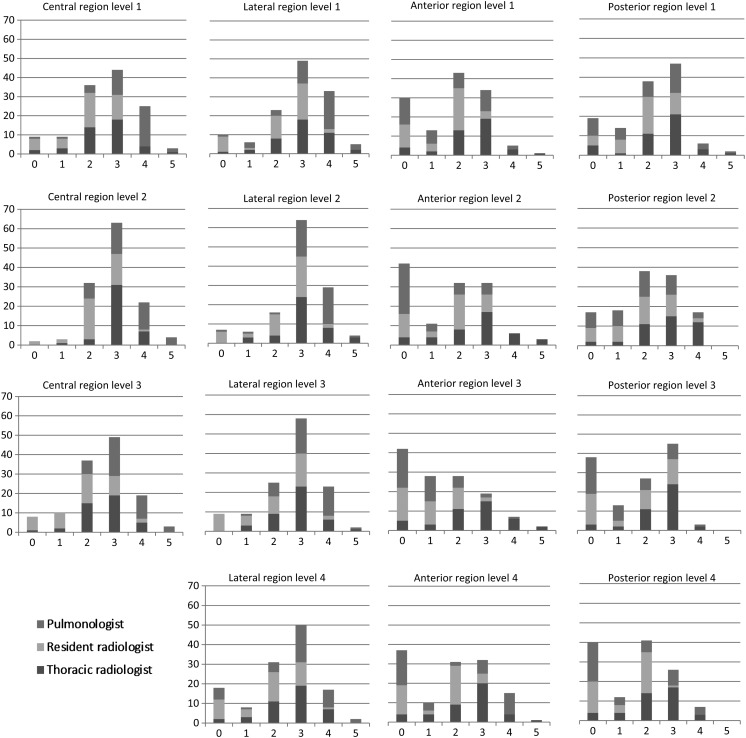
Histograms of reported visibility according to location and observer. Results from left and right lungs are pooled. The *x*-axis represents visibility rating and the *y*-axis the number of structures.

**Figure 5.**
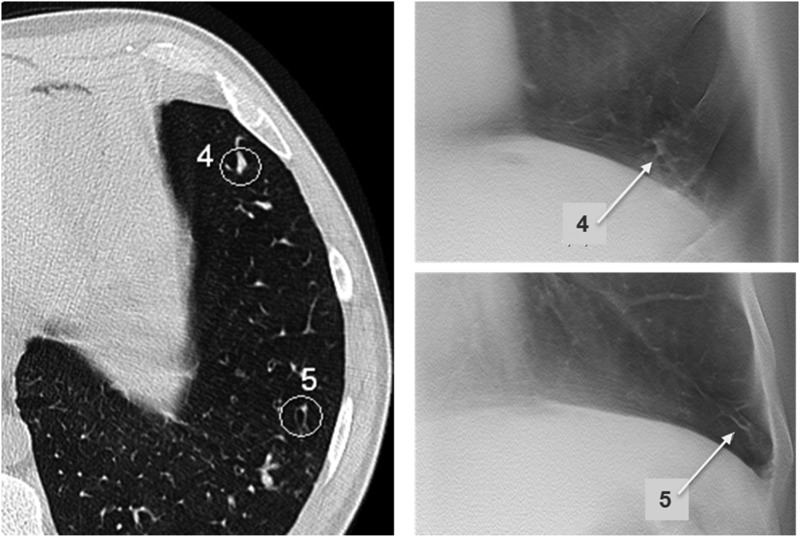
An illustration of the visibility of structures in the lower parts of the lower lobes surrounding the diaphragm. Structures in the lateral parts of the lungs are better visualised than structures in the anterior and posterior aspects. The structure marked 4 is a peripheral mucus plugging located in the anterior region of the lung behind a rib. The experienced radiologist reported visibility as equal to CT (Grade 3), the resident radiologist stated inferior visibility (Grade 2) and the pulmonologist assessed the structure as not visible (Grade 0). The structure marked 5 is a peripheral bronchiectasis lateral to the diaphragm, reported visibility was 3 (resident radiologist), 4 (experienced radiologist) and 5 (pulmonologist).

**Figure 6.**
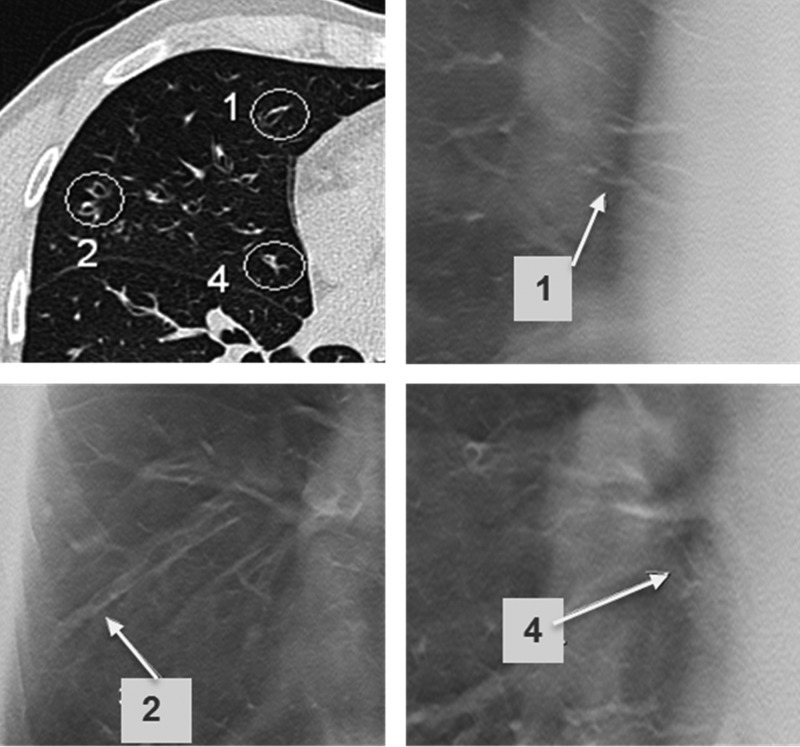
Examples of peripherally and centrally located dilated bronchi in the right middle lobe. Structure 1 is a peripheral bronchiectasis in the anterior region. Visibility score ranged from not visible (0, pulmonologist) to equal visibility (3, experienced radiologist). Structure 2 is a peripheral bronchiectasis with distal mucus plugging. All observers reported visibility superior to CT (Grade 4). Structure 4 is a central bronchus with thickened wall located close to the heart and great vessels. All observers assessed visibility as slightly inferior to CT (Grade 2).

Visibility ratings were similar in both lungs, and ratings from corresponding regions were therefore pooled in the statistical analyses and histograms. In DTS, structures in the central and peripheral lateral regions of the lungs received higher ratings compared with that of the peripheral anterior and posterior regions (*p* ≤ 0.001). Among the ratings of lateral structures, 67 % were equal or superior to CT, and the corresponding result for central structures was 61 %. There were no significant differences in visibility according to anatomical level. Histograms of visibility according to location and observer are given in Figure 4. Images exemplifying differences in visibility according to anatomical location are given in Figures 5 and 6.

### Dependency on observer experience

**Figure 7.**
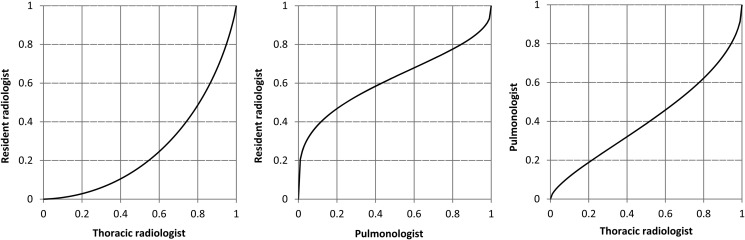
VGC curves illustrating the inter-observer variability of visibility ratings. The curve to the left compares visibility ratings between the resident radiologist and the experienced thoracic radiologist. The area under the curve is <0.5, indicating an overall higher visibility rating by the thoracic radiologist (*p* ≤ 0.001). The shape of the curve illustrates that the two observers used a similar range of ratings, but that the ratings were given at different levels of the grading scale. On the other hand, the S-shapes of the curves in the middle and to the right indicate differences in the range of visibility rating between the pulmonologist and the two radiologists.

VGC curves illustrating inter-observer variability of visibility ratings are presented in Figure 7. The experienced thoracic radiologist reported the visibility in DTS compared with CT higher than the two less experienced observers (*p* ≤ 0.01). The shape of the VGC curve for comparison between the experienced and inexperienced radiologists indicates that these two observers used a similar range of ratings, but that the ratings were given at different levels of the grading scale. It seems plausible that the experienced observer of this study required less details and/or contrast to assess the visibility in DTS as equal to CT. The S-shaped VGC curves for the comparisons between the pulmonologist and the two radiologists indicate differences in the rating range; the pulmonologist was more likely to use the extreme values of the scale (not visible and significantly improved visibility) in DTS compared with CT.

## DISCUSSION

The results of this study indicate impaired visibility of small anatomical structures in DTS when performing a head-to-head comparison with CT. Furthermore, the visibility in DTS was dependent on both anatomical location and observer experience. By direct comparison between the two modalities, the perceived visibility in DTS was equal to that in CT for 34 %, inferior to that in CT for 52 % and superior to that in CT for 14 % of the ratings. The visibility for DTS was significantly higher in the central and peripheral lateral regions than that in the anterior and posterior peripheral regions. The most experienced observer had significantly higher visibility ratings compared with the two less experienced observers.

Previous studies^(34, 35)^ have reported motion artefacts and limited depth resolution as main causes of reduced visibility in DTS, although those studies were not specifically designed to cover the correlation between visibility and anatomical location. The present study was designed to address problematic areas; the limited depth resolution was challenged in areas with parenchymal structures adjacent to dense objects such as clavicles, ribs, sternum, great vessels, heart and diaphragm, and motion artefacts were primarily visualised around the diaphragm. Intravenous devices like port-a-cath (48 % of the patients) and parenchymal opacities also contributed to artefacts in this patient group.

The results indicate that visibility is mostly affected by the limited depth resolution, leading to lower visibility of structures adjacent (anteriorly or posteriorly) to high-contrast objects. Structures further away from the focus plane are less disturbing since outlines become more blurred and the contrast decreases^(11)^. Ripple artefacts, seen as lines parallel to the diaphragm, were not considered to affect visibility in this study material. DTS images were well inspired, and estimated lung volume was slightly increased in comparison with estimates based on CT, which could be explained by the differences in position; DTS is performed in standing and CT in supine position. Hence, there was no indication that differences in inspiration would affect the visibility of assessed structures.

Previous studies have suggested DTS as an alternative to CT in the radiological monitoring of patients with CF^(9, 35, 36)^. This suggestion is mainly based on the superior sensitivity in detecting pathology compared with CXR, and the substantially lower radiation dose compared with CT. The present study, however, raises concerns regarding the proportion of ratings with visibility assessed as inferior to the gold standard reference CT, and whether replacing CT with DTS could affect general and focal assessment of disease severity, and thereby possibly delay diagnosis or treatment of progression of pathological changes of CF.

General and severe disease can often be diagnosed with functional test and laboratory analyses. Identifying subtle or focal pathology is one of the key roles in imaging of CF, and it is therefore crucial that the modality of choice is able to reveal these lesions^(18)^. The present study involves discrete pathology in problematic regions, where structures identified in CT were reported as barely or not visible in DTS in 25 % of the ratings. Small airway obstruction, seen as air trapping on expiratory CT images, is a central pathology of CF with good correspondence to lung function tests^(37–39)^, and these changes cannot be visualised on the inspiratory DTS images. Ground glass opacity is one of the less common pulmonary changes of CF, and previous studies of artificial lung nodules have indicated inferior detectability of ground glass opacities on DTS compared with CT^(40)^. The present study only included two ground glass opacities, and no conclusion can therefore be drawn regarding visibility of these changes.

### Observer experience and learning aspects

Asplund *et al.*^(41)^ performed a study where detection of lung nodules in DTS was significantly increased among inexperienced observers after learning with feedback. From this perspective, having only one experienced observer naturally provides speculative results regarding dependency on observer experience as perceived visibility might have been improved by a learning session, or by including only experienced radiologists, or experienced CF physicians as observers. On the other hand, the aim of this study was to investigate how perceived visibility was affected by experience and background, and the results indicate that the participating clinician had a tendency to polarise visibility of structures so that they were either classified as not visible, or as having superior visibility in DTS compared with CT. In contrast, the ratings of the two radiologists were in a narrower spectrum of the scale. The experienced thoracic radiologist had been trained in detection of the subtle gray-scale differences of CXR and DTS, and the resident radiologist preferred the high-contrast images of CT, which may explain the overall higher visibility ratings of the experienced thoracic radiologist. In the statistical evaluation of the comparison between anatomical locations, a random-reader analysis was used in order to take the variation between the observers into account and to achieve a generalisation of the results to a population of observers.

### Radiation exposure

Life-time risk of radiation-induced cancer is difficult to assess. Some studies^(21, 42)^ consider the risk related to biennial low-dose CT in this patient group with life-limiting disease to be small, and de González *et al.*^(43)^ estimated cumulative risk of radiation-induced cancer from annual CT to <0.5 %. However, the fact that median survival age for patients with CF has increased over the past two decades from ∼30 y to ∼50 y in industrial countries^(44)^ emphasises the importance to consider low-dose alternatives to CT according to the principle ‘as low as reasonably achievable’^(45)^ for this young patient population. Furthermore, the high sensitivity of CT is associated with the highest percentage of incidental findings among the imaging modalities^(46, 47)^, potentially exposing the patient to unnecessary additional radiation in the follow-up of a benign lesion.

Both CT and DTS are currently undergoing dose optimisation, and low-dose CT protocols below 1 mSv are available. Studies based on visibility of lung nodules indicate that DTS dose can be reduced by at least 50 % (<0.07 mSv) without compromising detectability^(48, 49)^. Further, replacing current standard surveillance combinations of bienniel or triennial CT and CXR with annual DTS could possibly increase chances of early detection and treatment of progressive disease, at substantially lower radiation doses. A radiological department in Sweden^(50)^ has experience in using annual DTS as the standard modality for surveillance of CF in children. The children typically cooperate to the 10-s breath hold from the age of 5 y. CT is only performed in younger children and in selected cases where DTS is not possible or insufficient. Finally, magnetic resonance imaging stands out as an emerging and promising high-performance modality that has the ability to depict pulmonary changes of CF without any ionising radiation^(16, 51, 52)^.

### Study limitations

The present study is based on a relatively small sample size. Each of the three observers represents a different category of doctors evaluating these images; and having only one observer in each category compromises the possibility of generalising the results regarding how visibility is influenced by observer experience, and to a lesser extent the dependency on anatomical location. Furthermore, as the study was focussed on the visibility of structures according to anatomical location, no conclusion can be drawn regarding the visibility of specific pathological changes related to CF. Finally, the reference CT examinations were performed on different machines, but scan protocols were similar and there were no apparent differences in image quality.

## CONCLUSION

The results indicate that minor pathology can be difficult to visualise with DTS depending on location and observer experience. Central and peripheral lateral structures of relevance for monitoring of CF are generally well depicted by the new modality. Further research is required to investigate the clinical importance of detecting all minor pathology in the imaging surveillance of CF, and if training with feedback could increase the perceived visibility of these structures in tomosynthesis.

## FUNDING

This work was supported by grants from the Swedish Research Council (2011-488, 2013-3477), the Swedish Radiation Safety Authority (2012-2021, 2013-2982), the Swedish Federal Government under the LUA/ALF agreement (ALFGBG-136281, ALFGBG-428961) and the Health and Medical Care Committee of the Region Västra Götaland (VGFOUREG-81341, VGFOUREG-483951). Funding to pay the Open Access publication charges for this article was provided by the Swedish Research Council.
